# Individual and Combined Association between Unhealthy Lifestyle Behaviors and Body Weight Dissatisfaction in a Sample of Adolescents from Southern Brazil

**DOI:** 10.3390/children10050821

**Published:** 2023-04-30

**Authors:** Suellem Zanlorenci, Leticia Gonçalves, Tiago Rodrigues de Lima, Diego Augusto Santos Silva

**Affiliations:** Research Center in Kinanthropometry and Human Performance, Department of Physical Education, Sports Center, Federal University of Santa Catarina, University Campus—Trindade—n. 476, Florianópolis 88040-900, Santa Catarina, Brazil; suellemzan@gmail.com (S.Z.); leticia.g.2008@hotmail.com (L.G.); tiagopersonaltrainer@gmail.com (T.R.d.L.)

**Keywords:** health promotion, adolescent health, body weight, lifestyle

## Abstract

The individual and combined association between unhealthy lifestyle behaviors and body weight dissatisfaction in adolescents was investigated. This cross-sectional research used data from 676 students (348 female), aged between 14 and 19 years old (mean of 14.6 years old) from southern Brazil. Unhealthy lifestyle behaviors and body weight dissatisfaction were assessed through a questionnaire. Unhealthy lifestyle behaviors included smoking, excessive alcohol consumption, sedentary lifestyle, and poor diet, and were analyzed as individual factors and as combinations of behaviors. For males, smoking (OR: 2.6; 95% CI: 1.6–4.0) and the combination of smoking/excessive consumption of alcoholic beverages (OR: 2.5; 95% CI: 1.1–5.8) were directly associated with the desire to reduce body weight, whereas the combination of smoking/inadequate diet (OR: 1.3; 95% CI: 1.0–1.6) was associated with the desire to increase body weight. For females, the combinations of inadequate diet/physical inactivity (OR: 1.6; 95% CI: 1.0–2.5) and of smoking/excessive consumption of alcoholic beverages (OR: 1.9; 95% CI: 1.4–2.8) were directly associated with the desire to reduce body weight. The presence of simultaneous unhealthy lifestyle behaviors is associated with bodyweight dissatisfaction in adolescents.

## 1. Introduction

Puberty is seen as an intermediate stage between childhood and adulthood, marked by notable changes in the body, personality, and attitudes [1]. During this phase of life, adolescents experience body changes related to puberty (i.e., development of secondary sexual characteristics and growth acceleration), which in addition to the need to meet society’s expectations and impositions can represent a challenge for bodyweight perception [2]. In a context marked by bodily transformations and changing responsibilities and behavioral patterns, young people who cannot achieve certain physical characteristics (i.e., thinness for women, and lean mass and muscle for men) [2,3], may be susceptible to developing bodyweight dissatisfaction [4].

The estimated prevalence of bodyweight dissatisfaction in adolescents (11 to 15 years old) from developed countries ranges from 51.0% in the USA to 55.2% in Italy for females, and 37.7% in the USA to 39.9% in Italy for males [5]. In Brazil, the estimated prevalence of bodyweight dissatisfaction in adolescents (12 to 17 years old) of either sex is 45.0% [6]. In addition to sex (females were identified as being more prone to bodyweight dissatisfaction compared with males) [7], high income level [8], higher age group [7], and being overweight [7] were aspects directly associated with bodyweight dissatisfaction among adolescents.

In addition to the sociodemographic aspects highlighted above, there is growing evidence showing the direct relationship between unhealthy lifestyle behaviors (e.g., inadequate eating habits, physical inactivity, smoking, alcohol consumption) and bodyweight dissatisfaction [2,4,7,9,10]. Inadequate eating habits (i.e., foods with high caloric density, rich in sodium, fat, and sugar) [4] and physical inactivity [11] contribute to an individual’s energy balance, leading to an increase in body weight, which is ultimately connected to feeling dissatisfied with one’s body weight [4,11]. Smoking has been described as a strategy to control or reduce body weight, since the feeling of satiety resulting from nicotine levels in the blood contributes to adolescents remaining fasting for a large part of the day or eating as little as possible [12]. In addition, alcohol consumption is directly related to bodyweight dissatisfaction, since adolescents who face changes in bodyweight perception may consider alcohol consumption as a strategy for bodyweight change, because alcohol can be used as a potential source of energy, which is associated with weight gain [13], or as a method of relieving emotional suffering due to bodyweight dissatisfaction (i.e., stress, anxiety, or anguish) [14].

Although unhealthy lifestyle behaviors have been recognized as being associated with bodyweight dissatisfaction, the synergistic effect attributed to the adoption of multiple unhealthy behaviors associated with body weight is not known. The simultaneous investigation of unhealthy lifestyle behaviors and association with bodyweight dissatisfaction is particularly relevant, considering that adolescents tend to adopt multiple behaviors at the same time, instead of adopting them individually [15]. In this sense, the results of the present study add information to the literature regarding the simultaneity of unhealthy lifestyle behaviors in association with bodyweight dissatisfaction, which is one of the main differences between this study and others previously published [7,12,16].

The aim of the present study was to investigate the individual and combined associations between unhealthy lifestyle behaviors (inadequate diet, alcohol consumption, smoking, and physical inactivity) and bodyweight dissatisfaction among adolescents.

## 2. Materials and Methods

### 2.1. Study Design, Ethical Considerations, and Participants

This school-based epidemiological research with a cross-sectional design is linked to the macroproject “Brazilian Guide for the Assessment of Physical Fitness and Life Habits—Stage II”. This study was carried out in the city of São José/SC, southern Brazil. The study population consisted of 5411 high school students from state public schools in the city of São José, distributed through 11 eligible schools and 186 high school classes. The study’s comprehensive details are documented in the literature [17,18].

Sampling was carried out in two stages, initially stratified by school density (small, medium, and large schools), followed by the number of classes based on school grade and shift. With a 95% confidence interval, a 5% tolerable error, a 50% prevalence, a design effect of 1.5, and 20% added to account for possible losses, refusals, and confounding variables, a total of 1233 students were required. In 2019, 891 adolescents were evaluated from the selected public schools, with 676 students (75.9% of the total sample) providing all the information related to their satisfaction/dissatisfaction with their body weight and other variables included in this study.

The macroproject was approved by the Human Research Ethics Committees of the Federal University of Santa Catarina, with protocol No. 3.523.470. Participants under 18 years old were obligated to complete a free and informed consent form, which had to be signed by their parents or legal guardians. Additionally, participants were required to sign the consent form themselves.

During a break from their regular classes for research participation, the students responded individually to the study questionnaire and then underwent physical tests. Any uncertainties were addressed by the evaluators during the questionnaire administration. The evaluators were previously trained to ensure consistency in the testing procedures and data collection.

### 2.2. Outcome Variable: Body Weight Satisfaction/Dissatisfaction

A question derived from a validated questionnaire for the Brazilian population was used to assess body weight dissatisfaction (Youth Risk Behavior Surveillance System) [19]. The reproducibility of questions in the questionnaire is high (kappa agreement index = 0.84), which enables its application in epidemiological studies in Brazil [20]. See details in Supplementary Table S1.

### 2.3. Exposure Variables: Unhealthy Lifestyle Behaviors

Alcohol consumption and smoking were assessed using questions derived from the Youth Risk Behavior Surveillance System questionnaire [19] and were categorized according to the literature recommendations [21,22]. Detailed information is provided in Supplementary Table S1.

A questionnaire validated for the Brazilian population was used to assess the level of physical activity [20]. These research data were collected in 2019 and for this reason the physical activity guidelines published in 2010 were used [23]. See details in Supplementary Table S1.

With regard to eating habits, information regarding the adoption of inadequate diet during a typical week was collected (diet) [24]. Data were categorized according to the literature recommendations (Supplementary Table S1) [25].

Information regarding the adoption of unhealthy lifestyle behaviors (inadequate diet, alcohol consumption, smoking, and physical inactivity) were analyzed as possible aspects associated with bodyweight satisfaction/dissatisfaction. In this context, these unhealthy behaviors were investigated as individual exposure variables, combinations of two unhealthy lifestyle behaviors, and the number of unhealthy behaviors simultaneously adopted by each adolescent (0–4 unhealthy lifestyle behaviors) in association with bodyweight satisfaction/dissatisfaction. Considering that fewer than 4% of male adolescents and 7% of female adolescents had an absence of unhealthy lifestyle behaviors, category “0” was incorporated into category “1” when considering numbers of unhealthy lifestyle behaviors.

### 2.4. Characterization and Control Variables

Anthropometric measurements (height and body mass) were performed according to literature recommendations [26]. BMI was calculated from the measurements of weight and height. The cut-points used were determined by BMI z-score, considering standard deviations according to sex and age [27].

Sociodemographic information collected and analyzed included sex, age, and economic level. Economic level was determined from a questionnaire used to assess the purchasing power of Brazilian families [28,29].

Sexual maturation was assessed according to Tanner’s criteria using stages that indicate the development stage of pubic hair (female) and genitalia size (male) [30]. These stages contained images related to the five stages of maturation, and were categorized according to the literature recommendations [31]. Information regarding the categorization and description of the investigated variables can be viewed in full detail in Supplementary Table S1.

### 2.5. Data Analysis

Descriptive and inferential statistics were used to describe the analyzed information. Categorical variables are presented as percentages (%) with corresponding 95% confidence intervals (95% CI). In addition, the chi-square test for heterogeneity and Fisher’s exact test were used to identify possible differences between groups according to satisfaction/dissatisfaction with body weight. Considering the determinant relationship that gender plays in the individual perception of body weight and the different factors associated with satisfaction/dissatisfaction with body weight [10], analyses were conducted and stratified according to sex.

Multinomial logistic regression models with estimation of odds ratio (OR) and 95% CI were used (with “satisfied with body weight” category as reference) to test the association between bodyweight dissatisfaction and each of the variables related to unhealthy lifestyle behaviors. Age, economic level, BMI, and sexual maturation were included as control variables for each multinomial regression model adopted to investigate the possible association with variables related to unhealthy lifestyle behaviors. In addition, with the exception of variables related to unhealthy behaviors or lifestyle behaviors tested for exposure in multivariate models, the remaining variables related to unhealthy lifestyle behaviors were added as control variables. For example, when investigating the association between bodyweight dissatisfaction and inadequate diet, smoking and alcohol consumption were also included as control variables in addition to age, economic level, BMI and sexual maturation, regardless of levels of statistical significance in association with the outcomes.

Analyses were performed considering sample weights and study design. Data analysis was performed using the Stata software (version 16.0, Stata Corp LP, College Station, TX, USA), with *p* value < 0.05 considered statistically significant.

## 3. Results

In this study, 676 adolescents were assessed having provided complete information on all monitored outcomes. Of the total number of females (*n* = 348), 19.9% were satisfied with their body weight, 24.9% would have liked to increase their body weight and 55.1% would have liked to reduce their body weight. Of the total number of males (*n* = 328), 32.8% were satisfied with their body weight, 32.7% would have liked to increase their body weight and 34.5% would have liked to reduce their body weight (information not described in figures or tables).

General sample information according to bodyweight satisfaction/dissatisfaction is available in Supplementary Table S2 (females) and Supplementary Table S3 (males). Greater proportions of adolescents of both sexes who were satisfied with their body weight and/or who would have liked to increase their body weight had eutrophic weight status, while greater number of adolescents (males and females) who would have liked to reduce their body weight were overweight/obese (Supplementary Tables S2 and S3).

Crude association results indicated that, among females, alcohol consumption (OR: 1.6; 95% CI: 1.2–2.3), the combination of inadequate diet/physical inactivity (OR: 1.7; 95% CI: 0.6–5.2), and smoking/alcohol consumption (OR: 2.1; 95% CI: 1.4–3.2) were aspects directly associated with the desire to increase body weight. In the adjusted analysis, the results indicated that, among females, the combination of inadequate diet/physical inactivity (OR: 1.6; 95% CI: 1.0–2.5) and the combination of smoking/excessive consumption of alcoholic beverages (OR: 1.9; 95% CI: 1.4–2.8) were aspects directly associated with the desire to increase body weight (Table 1).

Crude association results indicated that among males, inadequate diet (OR: 1.2; 95% CI: 1.0–1.7) was directly associated with the desire to increase body weight. Furthermore, smoking (OR: 1.9; 95% CI: 1.2–2.9) was directly associated with the desire to reduce body weight. In the adjusted analysis, the results indicated that, among males, smoking (OR: 2.6; 95% CI: 1.6–4.0) and the combination of smoking/excessive alcohol consumption (OR: 2.5; 95% CI: 1.1–5.8) were associated with the desire to reduce body weight. Furthermore, among males, the combination of inadequate diet/smoking (OR: 1.3; 95% CI: 1.0–1.6) was associated with the desire to increase body weight (Table 2).

When analyzing the number of unhealthy lifestyle habits adopted by the same individual, adherence to four healthy lifestyle habits simultaneously was not associated with bodyweight dissatisfaction (Table 3).

## 4. Discussion

The results of this study indicate that, among females, the combination of unhealthy lifestyle behaviors, inadequate diet/physical inactivity, and smoking/excessive consumption of alcoholic beverages were aspects directly associated with the desire to reduce body weight. Among males, smoking and the combination of smoking/excessive consumption of alcoholic beverages were directly associated with the desire to reduce body weight. In addition, the combination of inadequate diet/smoking was directly associated with the desire to increase body weight in males.

It is hypothesized that the relationship between the simultaneity of unhealthy lifestyle behaviors and bodyweight dissatisfaction may be related to the synergistic action of each unhealthy lifestyle behavior including (1) the adoption of unbalanced diet (i.e., inadequate consumption of cereals, grains, fruits, vegetables, meat and derivatives, and milk and dairy products), which can result in increased consumption of high-calorie foods compared with those with lower caloric value [2,7], contributing to increased weight status and bodyweight dissatisfaction [4]. (2) The practice of physical activity is an important modulator of maintaining energy balance, as it contributes to increasing calorie/energy expenditure. In this way, it is possible that less engagement in physical activities may contribute to a positive energy balance and consequently to the risk of obesity, being directly associated with bodyweight dissatisfaction [4]. (3) The interrelation between smoking and bodyweight dissatisfaction has been plausibly related to the effects that substances present in tobacco exert on the regulation of the central nervous system, especially for satiety and hunger control [12]. (4) It is speculated that alcohol consumption and smoking are related to the need to reduce depressive symptoms and eating disorders that are more frequently experienced by adolescents dissatisfied with their body weight [13].

With regard to the results verified for females, in addition to the direct impact attributed to inadequate diet and reduced time performing physical activities, the context in which the adolescent is situated can provide numerous factors that contribute to the simultaneous adoption of these habits [15]. This is because the amount of time spent by adolescents in front of electronic devices and screens is increasing, constituting a favorable environment both for the consumption of easily accessible foods such as “fast foods” and less time for the practice of physical activities [4]. Regarding the combination of smoking/excessive consumption of alcoholic beverages, which was directly associated with the desire to reduce body weight in adolescents of either sex, the justification for adopting such unhealthy behaviors may be related to vulnerability to nicotine addiction and the capability of modulating the central nervous system toward the need to consume products such as alcohol that can produce pleasurable sensations [32].

With regard to results identified exclusively for males, tobacco use was directly associated with the desire to reduce body weight, while the combination of inadequate diet/smoking habits was directly associated with the desire to increase weight. Results different from those of the present study were reported in a study carried out with male and female adolescents (n = 1019/46.6% male) from Rio de Janeiro, Brazil, in which adolescents whose dissatisfaction with their body weight was due to the desire to reduce body weight were less likely to smoke (OR = 0.36, 95% CI 0.13–0.98) [33]. Although smoking was individually directly associated with the desire to reduce body weight in this study, the combination of smoking with inadequate diet was directly associated with the desire to increase body weight. Considering that diet is the major determinant of bodyweight variability [34], it is speculated that the results found may be related to the greater influence exerted by inadequate diet compared with smoking on the desire to reduce or increase body weight. Additionally, it is speculated that aspects not investigated in the present study and likely to moderate the interrelationship between bodyweight dissatisfaction, inadequate diet, and smoking, such as the presence of symptoms suggestive of eating disorders [13,35], psychological factors (i.e., self-esteem and self-concept) [35], and social factors (i.e., comparisons of physical appearance) [36] may contribute to the fact that smoking was associated with the desire to reduce body weight when not combined with other unhealthy lifestyle habits and was associated exclusively in male adolescents with the desire to increase body weight when combined with another unhealthy lifestyle habit (inadequate diet).

The present study has strengths that should be highlighted, including the adoption of the instrument used to obtain information regarding outcomes and exposure, which was validated for the investigated population [20,24,37]. Another positive aspect in this context was the sampling design (complex sampling), attributing different weights to the probabilistic selection of sample elements, which considered the school size and the sample weight of each student, making estimates representative of the population [36]. However, some limitations should also be highlighted: (1) the cross-sectional design of the study, which did not allow causality and temporality to be established for the tested associations; (2) the insufficient number of individuals in some groups, which reduced the power available to test the associations of interest; (3) the questionnaire used in the present study to acquire information regarding physical activity, which made it impossible to classify the level of physical activity of adolescents according to the most recent guidelines (2020) [38]; (4) the lack of investigation of differences between BMI associated with bodyweight dissatisfaction. Although BMI is directly related to bodyweight dissatisfaction, it is necessary to emphasize that the individual’s perception of weight status is not limited to a weight status indicator, since it is possible that adolescents may want to lose weight even though they have an appropriate BMI, or opposite cases in which the adolescent can declare themselves as having adequate weight even if their BMI suggests overweight or obesity. However, it is noteworthy that the physical activity classification in the present study [23] was in accordance with current physical activity recommendations for the year in which the study was conducted.

## 5. Conclusions

The results of this study indicated that, for females, the combination of inadequate diet/physical inactivity and combination of smoking/excessive consumption of alcoholic beverages were aspects directly associated with the desire to increase body weight. For males, smoking and the combination of smoking/excessive consumption of alcoholic beverages were directly associated with the desire to reduce body weight, and the combination of smoking/inadequate diet was associated with the desire to increase body weight.

## Figures and Tables

**Table 1:** Crude and adjusted multinomial logistic regression analysis between unhealthy lifestyle habits and females’ bodyweight satisfaction/would like to increase body weight and/or would like to reduce body weight.

	Crude Analysis						Adjusted Analysis					
	Would Like to Increase Body Weight			Would Like to Reduce Body Weight			Would Like to Increase Body Weight			Would Like to Reduce Body Weight		
Variables	OR	95% CI	*p*-Value	OR	95% CI	*p*-Value	OR	95% CI	*p*-Value	OR	95% CI	*p*-Value
**Inadequate diet ^a^**												
Adequate	1			1			1			1		
Inadequate	1.1	(1.7–7.2)	0.86	6.6	(3.3–13.3)	0.12	0.9	(1.2–8.1)	0.98	0.7	(0.4–11.1)	0.64
**Physically active ^b^**												
Physical activity	1			1			1			1		
Physically inactive	0.7	(0.2–2.8)	0.38	1.2	(0.2–5.6)	0.86	0.7	(0.2–2.3)	0.37	1.5	(0.1–2.8)	0.78
**Smoking ^c^**												
Not exposed	1			1			1			1		
Exposed	1.6	(0.7–3.5)	0.13	1.2	(0.5–2.9)	0.52	1.3	(0.3–5.8)	0.49	0.8	(0.2–3.5)	0.51
**Alcohol consumption ^d^**												
Not exposed	1			1			1			1		
Exposed	1.6	(1.2–2.3)	**0.02 ^†^**	1.4	(0.6–3.2)	0.25	1.5	(0.8–2.5)	0.09	1.5	(0.2–9.6)	0.47
**Inadequate diet × physically inactive ^e^**												
Adequate	1			1			1			1		
Inadequate	1.7	(0.6–5.2)	**<0.01 ** ^†^	2.0	(0.0–7.4)	0.74	1.6	(1.0–2.5)	**<0.01 ** ^†^	1.4	(0.1–2.8)	0.36
**Inadequate diet × smoking ^f^**												
Adequate	1			1			1			1		
Inadequate	1.3	(0.3–5.1)	0.53	0.7	(0.3–1.5)	0.18	1.2	(0.2–6.7)	0.72	0.7	(0.1–1.6)	0.70
**Inadequate diet × alcohol consumption ^g^**												
Adequate	1			1			1			1		
Inadequate	0.9	(0.2–3.4)	0.79	0.8	(0.3–2.0)	0.46	0.8	(0.2–2.8)	0.59	0.9	(0.2–4.9)	0.97
**Physically inactive × smoking ^h^**												
Adequate	1			1			1			1		
Inadequate	0.9	(0.8–1.2)	0.97	1.5	(0.2–8.6)	0.70	1.1	(0.1–1.2)	0.92	1.5	(0.1–6.5)	0.67
**Physically inactive × alcohol consumption ^i^**												
Adequate	1			1			1			1		
Inadequate	0.6	(0.1–3.9)	0.34	0.9	(0.2–4.9)	0.76	0.6	(0.1–6.8)	0.47	1.7	(0.1–8.4)	0.62
**Smoking × alcohol consumption ^j^**												
Adequate	1			1			1			1		
Inadequate	2.1	(1.4–3.2)	**0.02 ** ^†^	1.6	(0.4–6.4)	0.29	1.9	(1.4–2.8)	**0.01 ** ^†^	1.4	(0.2–8.1)	0.48

CI: Confidence interval, OR: odds ratio, ^†^ *p* < 0.05. ^a^: Results adjusted for body mass index, economic status, age, sexual maturation, alcohol, smoking, and physical activity; ^b^: Results adjusted for body mass index, economic status, age, sexual maturation, alcohol, diet, and physical activity; ^c^: Results adjusted for body mass index, economic status, age, sexual maturation, alcohol, diet, and physical activity; ^d^: Results adjusted for body mass index, economic status, age, sexual maturation, alcohol, smoking and physical activity; ^e^: Results adjusted for body mass index, economic status, age, sexual maturation, alcohol, and smoking; ^f^: Results adjusted for body mass index, economic status, age, sexual maturation, alcohol, and physical activity; ^g^: Results adjusted for body mass index, economic status, age, sexual maturation; smoking, and physical activity; ^h^: Results adjusted for body mass index, economic status, age, sexual maturation, alcohol, smoking, and diet; ^i^: Results adjusted for body mass index, economic status, age, sexual maturation, diet and physical activity; ^j^: Results adjusted for body mass index, economic status, age, sexual maturation, alcohol, diet, and physical activity.

**Table 2:** Crude and adjusted multinomial logistic regression analysis between unhealthy lifestyle habits and males’ bodyweight satisfaction/would like to increase body weight and/or would like to reduce body weight.

	Crude Analysis						Adjusted Analysis					
	Would Like to Increase Body Weight			Would Like to Reduce Body Weight			Would Like to Increase Body Weight			Would Like to Reduce Body Weight		
Variables	OR	95% CI	*p*-Value	OR	95% CI	*p*-Value	OR	95% CI	*p*-Value	OR	95% CI	*p*-Value
**Inadequate diet ^a^**												
Adequate	1			1			1			1		
Inadequate	1.2	(1.0–1.7)	**0.04 ^†^**	0.8	(0.2–3.4)	0.65	1.2	(0.7–2.0)	0.28	0.7	(0.5–1.1)	0.07
**Physically active ^b^**												
Physical activity	1			1			1			1		
Physically inactive	0.9	(0.3–2.9)	0.65	0.7	(0.1–5.7)	0.58	0.9	(0.4–1.8)	0.57	0.6	(0.0–2.3)	0.56
**Smoking ^c^**												
Not exposed	1			1			1			1		
Exposed	1.1	(0.4–2.7)	0.78	1.9	(1.2–2.9)	**0.02 ^†^**	0.9	(0.5–1.5)	0.71	2.6	(1.6–4.0)	**0.01 ^†^**
**Alcohol consumption ^d^**												
Not exposed	1			1			1			1		
Exposed	0.9	(0.8–5.1)	0.87	1.3	(0.9–1.9)	0.09	0.8	(0.1–6.5)	0.76	1.4	(0.6–3.5)	0.26
**Inadequate diet × physically inactive ^e^**												
Adequate	1			1			1			1		
Inadequate	0.7	(0.0–4.1)	0.82	0.5	(0.0–5.3)	0.69	0.6	(0.0–5.1)	0.81	0.5	(0.0–1.2)	0.62
**Inadequate diet × smoking ^f^**												
Adequate	1			1			1			1		
Inadequate	1.3	(0.7–2.2)	0.22	1.1	(0.7–1.8)	0.37	1.3	(1.0–1.6)	**0.04 ^†^**	0.9	(0.6–1.4)	0.53
**Inadequate diet × alcohol consumption ^g^**												
Adequate	1			1			1			1		
Inadequate	0.9	(0.1–7.1)	0.87	0.9	(0.7–1.2)	0.28	0.9	(0.1–6.4)	0.91	0.7	(0.1–6.5)	0.62
**Physically inactive × smoking ^h^**												
Adequate	1			1			1			1		
Inadequate	1.0	(0.2–6.4)	0.94	0.9	(0.2–5.1)	0.84	1.1	(0.3–3.9)	0.86	0.7	(0.0–2.9)	0.76
**Physically inactive × alcohol consumption ^i^**												
Adequate	1			1			1			1		
Inadequate	0.5	(0.1–4.1)	0.27	0.4	(0.0–6.7)	0.32	0.5	(0.1–2.2)	0.17	0.3	(0.0–1.3)	0.31
**Smoking × alcohol consumption ^j^**												
Adequate	1			1			1			1		
Inadequate	1.1	(0.3–4.9)	0.77	1.7	(0.9–3.5)	0.07	0.9	(0.1–6.6)	0.94	2.5	(1.1–5.8)	**0.04 ^†^**

CI: Confidence interval, OR: odds ratio, ^†^ *p* < 0.05. ^a^: Results adjusted for body mass index, economic status, age, sexual maturation, alcohol, smoking, and physical activity; ^b^: Results adjusted for body mass index, economic status, age, sexual maturation, alcohol, diet, and physical activity; ^c^: Results adjusted for body mass index, economic status, age, sexual maturation, alcohol, diet, and physical activity; ^d^: Results adjusted for body mass index, economic status, age, sexual maturation, alcohol, smoking, and physical activity; ^e^: Results adjusted for body mass index, economic status, age, sexual maturation, alcohol, and smoking; ^f^: Results adjusted for body mass index, economic status, age, sexual maturation, alcohol, and physical activity; ^g^: Results adjusted for body mass index, economic status, age, sexual maturation, smoking, and physical activity; ^h^: Results adjusted for body mass index, economic status, age, sexual maturation, alcohol, smoking, and diet; ^i^: Results adjusted for body mass index, economic status, age, sexual maturation, diet, and physical activity; ^j^: Results adjusted for body mass index, economic status, age, sexual maturation, alcohol, diet, and physical activity.

**Table 3:** Crude and adjusted multinomial logistic regression analysis between females and males’ bodyweight satisfaction/would like to increase body weight and/or would like to reduce body weight and unhealthy lifestyle habits.

	Crude Analysis						Adjusted Analysis					
	Would Like to Increase Body Weight			Would Like to Reduce Body Weight			Would Like to Increase Body Weight			Would Like to Reduce Body Weight		
Simultaneous Adoption of Unhealthy Lifestyle Habits	OR	95% CI	*p*-Value	OR	95% CI	*p*-Value	OR	95% CI	*p*-Value	OR	95% CI	*p*-Value
**Female **												
1 unhealthy habits lifestyle habits	1			1			1			1		
2 unhealthy habits lifestyle habits	0.3	(0.0–2.9)	0.14	0.5	(0.0–2.1)	0.68	0.3	(0.2–5.2)	0.22	0.9	(0.0–2.9)	0.97
3 unhealthy habits lifestyle habits	0.3	(0.0–2.5)	0.12	0.5	(0.0–6.6)	0.68	0.4	(0.0–1.8)	0.42	0.9	(0.1–3.0)	0.98
4 unhealthy habits lifestyle habits	0.2	(0.0–9.2)	0.22	0.3	(0.0–5.9)	0.57	0.4	(0.0–6.3)	0.64	0.6	(0.1–1.8)	0.89
**Male **												
1 unhealthy habits lifestyle habits	1			1			1			1		
2 unhealthy habits lifestyle habits	1.5	(0.1–2.7)	0.63	0.8	(0.1–8.6)	0.67	1.3	(0.2–8.6)	0.59	1.1	(0.0–8.9)	0.96
3 unhealthy habits lifestyle habits	1.3	(0.1–1.9)	0.73	1.0	(0.3–3.1)	0.97	1.2	(0.2–6.1)	0.62	1.1	(0.0–1.7)	0.94
4 unhealthy habits lifestyle habits	1.7	(0.2–15.8)	0.43	0.8	(0.0–3.4)	0.80	16	(0.4–6.5)	0.30	0.9	(0.0–1.9)	0.97

Results adjusted for body mass index, economic status, age, and sexual maturation; 95% CI: confidence interval, OR: odds ratio.

## Data Availability

All data can be requested from the authors if necessary.

## Supplementary Materials

The following supporting information can be downloaded at: https://www.mdpi.com/article/10.3390/children10050821/s1, Table S1: Table of variables; Table S2: Descriptive information of males adolescents, according to satisfaction/dissatisfaction with body weight. São José, SC, Brazil –2019; Table S3: Descriptive information of males adolescents, according to satisfaction/dissatisfaction with body weight. São José, SC, Brazil –2019. [20,21,22,23,25,26,27,28,29,30,31]

## References

[1] Tsai M.-C., Strong C., Lin C.Y. (2015). Effects of pubertal timing on deviant behaviors in Taiwan: A longitudinal analysis of 7th- to 12th-grade adolescents. J. Adolesc..

[2] Miranda V.P., Amorim P.R.S., Bastos R.R., Souza V.G.B., Faria E.R., Franceschini S.C.C., Teixeira P.C., Morais N.S., Priore S.E. (2021). Body image disorders associated with lifestyle and body composition of female adolescents. Public Health Nutr..

[3] McLean S.A., Paxton S.J. (2019). Body Image in the Context of Eating Disorders. Psychiatr. Clin. N. Am..

[4] Da Silva O.P., Guimarães J.M.N., Griep R.H., Melo E.C.P., Matos S.M.A., Molina M.D.C., Barreto S.M., Fonseca M.J.M. (2018). Association between Body Image Dissatisfaction and Self-Rated Health, as Mediated by Physical Activity and Eating Habits: Structural Equation Modelling in ELSA-Brasil. J. Environ. Res. Public Health.

[5] Al Sabbah H., Vereecken C.A., Elgar F.J., Nansel T., Aasvee A., Ojala K., Ahluwalia N., Maes L. (2009). Body weight dissatisfaction and communication with parents among adolescents in 24 countries: International cross-sectional survey. BMC Public Health.

[6] Moehlecke M., Blume C.A., Cureau F.V., Kieling C., Schaan B.D. (2020). Self-perceived body image, dissatisfaction with body weight and nutritional status of Brazilian adolescents: A nationwide study. J. Pediatr..

[7] Tebar W.R., Canhin D.S., Colognesi L.A., Ah Morano A.E.V., More D.T.C.S., Christofaro D.G.D. (2020). Body dissatisfaction and its association with domains of physical activity and of sedentary behavior in a sample of 15,632 adolescents. Int. J. Adolesc. Med. Health.

[8] Ben Ayed H., Yaich S., Jemaa M.B., Hmida M.B., Trigui M., Jedidi J., Sboui I., Karray R., Feki H., Mejdoub Y. (2019). What are the correlates of body image distortion and dissatisfaction among school-adolescents?. Int. J. Adolesc. Med. Health.

[9] Gonçalves L., Zanlorenci S., Borges L.L., de Lima T.R., Silva D.A.S. (2023). Body Weight Dissatisfaction and Health Risk Behaviors in Adolescents. Percept. Mot. Ski..

[10] Gonzaga I., Ribovski M., Claumann G.S., Folle A., Beltrame T.S., Laus M.F., Pelegrini A. (2023). Secular trends in body image dissatisfaction and associated factors among adolescents (2007–2017/2018). PLoS ONE.

[11] Morais N.S., Miranda V.P.N., Priore S.E. (2018). Imagem corporal de adolescentes do sexo feminino e sua associação à composição corporal e ao comportamento sedentário. Ciênc. Saúde Coletiva.

[12] Kilibarda B., Rakic J.G., Scekic S.M., Krstev S. (2020). Smoking as a weight control strategy of Serbian adolescents. Int. J. Public Health.

[13] Harrell Z.A., Slane J.D., Klump K.L. (2009). Predictors of alcohol problems in college women: The role of depressive symptoms, disordered eating, and family history of alcoholism. Addict. Behav..

[14] Rocha R.P., Galvão P.P.O., Sanchez Z.V.D.M., Rebouças L.N., Castro A.R.J., Santos L.E.S., Martins M.C., Pinheiro P.N.C., Vieira N.F.C. (2022). Insatisfação com a imagem corporal, uso de drogas e fatores associados entre adolescentes em três cidades brasileiras. Rev. Latino -Am. Enferm..

[15] Zappe J.G., Alves C.F., Dell’Aglio D.D. (2018). Comportamentos de Risco na Adolescência: Revisão Sistemática de Estudos Empíricos. Psic. Ver..

[16] Albawardi N.M., Al Tamimi A.A., Al Marzooqi M.A., Alrasheed L., Al-Hazzaa H.M. (2021). Associations of Body Dissatisfaction With Lifestyle Behaviors and Socio-Demographic Factors Among Saudi Females Attending Fitness Centers. Front. Psychol..

[17] De Lima T.R., Sui X., De Lima L.R.A., Silva D.A.S. (2021). Muscle strength and its association with cardiometabolic variables in adolescents: Does the expression of muscle strength values matter?. World J. Pediatr..

[18] De Lima T.R., Sui X., Silva D.A.S. (2021). Normalization of muscle strength measurements in the assessment of cardiometabolic risk factors in adolescents. J. Environ. Res. Public Health.

[19] Thomas J.R., Nelson J.K., Silverman S.J. (2009). Métodos de Pesquisa em Atividade Física.

[20] Guedes D.P., Lopes C.C. (2010). Validação da versão brasileira do Youth Risk Behavior Survey 2007. Rev. Saúde Pública.

[21] Géczy I., Saewyc E.M., Poon C., Homma Y. (2020). Health-Risk Behaviors and Protective Factors Among Adolescents in Rural British Columbia. J. Rural. Health.

[22] Gomez Y., Lisa M.C., Trivers K.F., Anic G., Morse A.L., Reissing C., Agaku I. (2020). Patterns of tobacco use and nicotine dependence among youth, United States, 2017–2018. Prev. Med..

[23] World Health Organization (2010). Global Recommendations on Physical Activity for Health.

[24] Rodriguez Añez C.R., Reis R.S., Petroski E.L. (2008). Versão brasileira do questionário” estilo de vida fantástico”: Tradução e validação para adultos jovens. Arq. Bras. Cardiol..

[25] Gledhill N. (2001). Introduction to the review papers pertaining to components of the Canadian Physical Activity, Fitness and Lifestyle Appraisal. Can. J. Appl. Physiol..

[26] Marfell-Jones M., Reilly T. (2005). Kinanthropometry VIII: Proceedings of the 8th International Conference of the International Society for the Advancement of Kinanthropometry (ISAK).

[27] World Health Organization (2007). Growth Reference Data for 5–19 Years. https://www.who.int/tools/growth-reference-data-for-5to19-years/indicators/bmi-for-age.

[28] Associação Brasileira de Empresas e Pesquisas (2010). Brazil Economic Classification Criterion.

[29] Critério Brasil-ABEP. https://www.abep.org/criterio-brasil.

[30] Quadros T.M.B., Gordia A.P., Silva L.R., Silva D.A.S. (2016). Inquérito epidemiológico em escolares: Determinantes e prevalência de fatores de risco cardiovascular. Cad. Saúde Pública.

[31] Tanner J.M. (1962). Growth at Adolescence.

[32] Benowitz N.L. (2010). Nicotine addiction. N. Engl. J. Med..

[33] Carvalho G.X., Nunes A.P.N., Moraes C.L., Veiga G.V. (2020). Insatisfação com a imagem corporal e fatores associados em adolescentes. Ciênc. Saúde Coletiva.

[34] Liberali R., Kupek E., Assis M.A.A. (2019). Dietary patterns and childhood obesity risk: A systematic review. Child. Obes..

[35] Oney C.N., Cole E.R., Sellers R.M. (2011). Racial identity and gender as moderators of the relationship between body image and self-esteem for African Americans. Sex Roles.

[36] Mitchell S.H., Petrie T.A., Greenleaf C.A., Martin S.B. (2012). Moderators of the internalization–body dissatisfaction relationship in middle school girls. Body Image.

[37] Szwarcwald C.L., Damacena G.N. (2008). Amostras complexas em inquéritos populacionais: Planejamento e implicações na análise estatística dos dados. Rev. Bras. Epidemiol..

[38] Bull F.C., Al Ansari SSBiddle S., Borodulin K., Buman M.P., Cardon C., Carty C., Chaput J.P., Chastin S., Chou R., Dempsey P.C. (2020). World Health Organization 2020 guidelines on physical activity and sedentary behaviour. Br. J. Sport. Med..

