# Supplementary material for: Individual and Combined Association between Unhealthy Lifestyle Behaviors and Body Weight Dissatisfaction in a Sample of Adolescents from Southern Brazil
Source: Children (Basel). 2023 Apr 30;10(5):821. doi: 10.3390/children10050821 (PMC10217292; doi:10.3390/children10050821)
Supplement: Supplementary file 1 [file children-10-00821-s001.zip › children-2354125-supplementary.pdf]

Supplementary Table S1. Table of variables

Variable	Assessment Method	Classification/Justification
Body weight satisfaction/dissatisfaction	Youth Risk Behavior Surveillance System: Adolescents answered the following question: "How would you describe your body weight?"[1].	Adolescents were classified as "would like to increase body weight" when they reported body weight as "well below expected" and "a little below expected". Those who reported body weight as "a little above expected" and "much above expected" were classified as "would like to reduce body weight". Adolescents who reported their body weight as "the expected weight" were classified as "satisfied with body weight".
Alcohol consumption	Youth Risk Behavior Surveillance System [1]: To classify students regarding alcohol consumption, those who consumed five or more doses of alcoholic beverages on a single occasion, at least once, in the last 30 days - according to the date on which the questionnaire was applied [2].	Were considered positive for alcohol consumption (inadequate habit regarding alcohol consumption) regardless of number of days (1 day or more) [2].
Smoking	Youth Risk Behavior Surveillance System[1]: "Do you smoke cigarettes?".	Adolescents who reported smoking, regardless of intensity or frequency, were considered to have inadequate habit regarding the use of cigarettes [3].
Physical activity	Youth Risk Behavior Surveillance System [1]	Adolescents who reported not performing physical activity for at least 60 minutes, seven days a week, were considered physically inactive. According to recommendations and through the same questionnaire, individuals aged 18 years or over were considered physically inactive when they did not perform at least 150 minutes of moderate-intensity physical activity over a period of one week, or did not perform at least 75 minutes of vigorous-intensity physical activity over a period of one week, or were not involved in an equivalent combination of moderate- and vigorous-intensity physical activity [4]. Data from this research were collected in 2019 and for this reason the physical activity guidelines published in 2010 was used [4].
Diet inadequate	The question used was: "Do you have a balanced diet?" [5].	The response options were the following: "almost never", "rarely", "sometimes", "with relative frequency" and "almost always". A balanced diet consists of cereals and grains (5 to 12 servings per day); fruits and vegetables (5 to 10 servings per day);

		meats and meat products (2 to 3 servings per day); and milk and dairy products (3 to 4 servings up to 16 years of age and 2 to 4 after 16 years of age) [5]. Response options “almost never”, “rarely” and “sometimes” were categorized as “inadequate” and “with relative frequency” and “almost always” were categorized as “adequate”.
Body mass	G-tech® digital scale (G-tech: Zhongshan, China), with resolution of 100g and capacity of 150kg.	Measurements (body mass) were performed according to literature recommendations [6].
Height	Sanny® (São Paulo, Brazil) stadiometer with tripod	Measurements (height) were performed according to literature recommendations [6].
Body mass index (BMI)	It is calculated by dividing weight (in kilograms) by height squared (in metres)	The cut-points used were determined by BMI z-score, considering standard deviations according to sex and age - “normal weight” or “overweight/ obesity” according to recommendations of the World Health Organization for age and sex [7].
Sex	Questionnaire	Male/Female
Age	Questionnaire	Continuous years
Economic level	Questionnaire used to assess the purchasing power of Brazilian families [8].	Classify families into a score ranging from “E” (low purchasing power) to “A” (high purchasing power). In the present study, purchasing power was categorized into “higher purchasing power” (categories “A” and “B”) and “lower purchasing power” (categories “C”, "D" and "E") [9].
Sexual maturation	Tanner's criteria [10]	To answer these questions, adolescents were separated into different rooms according to sex, and information on the interpretation of figures was provided by a research member of the same sex as the evaluated individual. Figure 1 represented the pre-pubertal stage, figures 2-4 represented the pubertal stage, and figure 5 the post-pubertal stage. In the present study, adolescents of both sexes were classified as “pre-pubertal”, “pubertal” and “post-pubertal” [11].

References:
[1] Guedes, D. P., Lopes, C.C. Validação da versão brasileira do Youth Risk Behavior Survey 2007. *Rev Saúde Pública* **2010**, v. 44, p. 840–850. https://doi.org/10.1590/S0034-89102010000500009.
[2] Géczy, I., Saewyc, E.M., Poon, C., Homma, Y. Health-Risk Behaviors and Protective Factors Among Adolescents in Rural British Columbia. *J Rural Health* **2020**, v. 36, n. 1, p. 65–76. https://doi.org/10.1111/jrh.12389.
[3] Gomez, Y., Lisa, M.C., Trivers, K.F., Anic, G., Morse, A.L., Reissing, C., Agaku, I. Patterns of tobacco use and nicotine dependence among youth, United States, 2017–2018. *Prev Med* **2020**, v. 141, p. 106284. https://doi.org/10.1016/j.ypmed.2020.106284.
[4] World Health Organization. Global Recommendations on Physical Activity for Health; World Health Organization, 2010; ISBN 92-4-159997-9.

[5] Gledhill, N. Introduction to the review papers pertaining to components of the Canadian Physical Activity, Fitness and Lifestyle Appraisal. *Can j appl physiol* **2001**, v. 26, n. 2, p. 157–160. https://doi.org/10.1139/h01-011.
[6] Marfell-Jones, M., Reilly, T(2005) Kinanthropometry VIII: Proceedings of the 8th International Conference of the International Society for the Advancement of Kinanthropometry (ISAK). [s.l.] Routledge.
[7] World Health Organization. Growth reference data for 5-19 years. 2007. Available in: http://www. who. int/growthref/en, 2016. Access in August 30,2022.
[8] Associação Brasileira de Empresas e Pesquisas. Brazil Economic Classification Criterion. 2010.
[9] Critério Brasil - ABEP. Available in: <https://www.abep.org/criterio-brasil>. Access in: August 29,2022.
[10] Quadros, T. M. B., Gordia, A.P., Silva, L.R., Silva, D.A.S. Inquérito epidemiológico em escolares: determinantes e prevalência de fatores de risco cardiovascular. *Cad Saúde Pública* **2016**, v. 32, n 2. https://doi.org/10.1590/0102-311X00181514.
[11] Tanner, J. M. Growth at adolescence. 1962.

Supplementary Table S2. Descriptive information of males adolescents, according to satisfaction/dissatisfaction with body weight. São José, SC, Brazil –2019.

	Satisfied with body weight		Would like to increase body weight		Would like to reduce body weight		*p*-value
**Variables**	**n**	**% (95%CI)**	**n**	**% (95%CI)**	**n**	**% (95%CI)**	
**Age**							0.87
14-16 years	46	33.4 (14.6-59.6)	44	30.4 (12.8-56.5)	49	36.2 (33.3-39.4)	
17-19 years	59	32.3 (20.8-46.5)	65	34.4 (22.1-49.3)	65	33.3 (26.5-40.8)	
**Economic Level**							0.22
Higher purchasing power	32	31.7 (0.4-82.4)	43	38.9 (20.3-61.5)	34	29.3 (0.9-62.5)	
Lower purchasing power	73	33.5 (31.2-35.8)	65	29.2 (14.3-50.6)	80	37.3 (22.7-54.7)	
**BMI***							**<0.01†**
Normal Wheigt	88	36.8 (34.3-39.4)	105	43.6 (21.5-68.6)	44	19.6 (4.9-54.2)	
Overweight/Obesity	17	22.3 (0.0-91.6)	4	4.8 (0.1-18.5)	70	72.9 (72.7-98.9)	
**Sexual Maturation**							0.37
Prepubescent	13	35.9 (22.8-51.7)	10	33.1 (11.7-64.8)	11	30.9 (17.0-49.4)	
Pubescent	74	34.8 (20.3-52.8)	75	32.9 (11.6-64.6)	71	32.3 (21.2-45.8)	
Post pubertal	18	25.6 (0.0-99.3)	23	31.2 (9.3-66.5)	32	43.4 (26.5-95.6)	
**Diet Inadequate**							0.45
Adequate	17	32.6 (9.9-67.9)	15	28.1 (22.2-34.7)	23	39.3 (17.5-66.5)	
Inadequate	88	32.9 (19.7-49.6)	93	33.4 (15.8-57.2)	91	33.6 (27.2-40.8)	
**Physically Active**							0.39
Physical activity	10	28.6 (9.7-60.0)	11	32.1 (11.7-62.8)	17	39.3 (6.1-86.5)	
Physically inactive	95	33.5 (18.9-52.1)	97	32.6 (13.3-60.3)	97	33.9 (26.3-42.6)	
**Smoking**							0.13
Not exposed	91	34.3 (19.2-53.5)	92	33.6 (19.6-51.3)	88	32.0 (29.8-34.3)	
Exposed	14	26.2 (9.4-54.8)	16	27.4 (8.3-61.4)	26	46.4 (36.0-57.1)	
**Alcohol consumption**							0.39
Not exposed	67	33.8 (24.1-45.2)	67	34.3 (27.8-41.5)	63	31.8 (26.8-37.3)	
Exposed	38	31.5 (10.2-65.1)	41	29.8 (6.9-70.9)	51	38.7 (30.9-46.9)	
**Inadequate Diet x Physically inactive ***							0.88
Adequate	1	21.6 (2.5-99.9)	2	31.2 (0.0-99.9)	3	47.2 (0.0-99.9)	
Inadequate	104	33.1 (19.2-50.7)	106	32.5 (17.3-52.7)	111	34.4 (30.9-38.0)	
**Inadequate Diet x Smoking**							0.79
Adequate	16	36.2 (35.8-89.7)	13	29.5 (17.8-44.6)	16	34.3 (5.5-82.4)	
Inadequate	89	32.3 (17.2-52.5)	95	33.0 (15.8-56.5)	98	34.6 (30.3-39.2)	
**Inadequate Diet x Alcohol consumption**							0.85
Adequate	10	31.1 (26.9-35.7)	10	33.2 (16.4-55.7)	13	35.7 (19.9-55.4)	
Inadequate	95	33.1 (16.3-55.6)	98	32.4 (14.3-58.1)	101	34.5 (31.0-38.1)	
**Physically inactive x Smoking**							0.60
Adequate	10	32.3 (16.4-53.7)	9	31.0 (8.2-69.4)	14	36.7 (5.4-85.6)	

Inadequate	95	32.9 (17.8-52.8)	99	32.7 (13.5-60.3)	10<br>0	34.3 (27.6-41.8)	
**Physically inactive x Alcohol consumption***							0.23
Adequate	4	19.0 (6.3-44.9)	7	38.1 (32.0-91.9)	11	42.8 (20.0-96.5)	
Inadequate	10<br>1	33.9 (18.1-54.4)	10<br>1	32.1 (12.6-60.7)	10<br>3	33.9 (27.1-41.6)	
**Smoking x Alcohol consumption**							0.06
Adequate	66	36.2 (26.7-46.9)	62	34.2 (25.5-44.1)	54	29.6 (27.7-31.6)	
Inadequate	39	28.9 (8.9-62.7)	46	30.5 (9.4-64.8)	60	40.7 (35.2-46.5)	
**Simultaneous adoption of unhealthy lifestyle behaviors***							0.72
1	3	34.8 (1.8-99.9)	3	24.5 (0.0-99.1)	5	40.7 (0.0-99.9)	
2	25	34.3 (15.9-59.2)	25	35.1 (29.1-41.6)	23	30.6 (15.4-51.6)	
3	50	32.3 (20.9-46.3)	47	28.9 (14.9-48.5)	61	38.7 (33.9-43.6)	
4	27	32.4 (17.9-51.2)	33	37.9 (11.9-73.3)	25	29.7 (14.4-51.4)	

Chi-square test of heterogeneity,*:Fisher's exact test, n: number or frequency, %: greater, BMI: body mass index, CI95%: Confidence interval,OR: Odds ratio, †: $p<0.05$.

Supplementary Table S3. Descriptive information of males adolescents, according to satisfaction/dissatisfaction with body weight. São José, SC, Brazil –2019.

	Satisfied with body weight		Would like to increase body weight		Would like to reduce body weight		*p*-value
**Variables**	**n**	**% (95%CI)**	**n**	**% (95%CI)**	**n**	**% (95%CI)**	
**Age**							0.87
14-16 years	46	33.4 (14.6-59.6)	44	30.4 (12.8-56.5)	49	36.2 (33.3-39.4)	
17-19 years	59	32.3 (20.8-46.5)	65	34.4 (22.1-49.3)	65	33.3 (26.5-40.8)	
**Economic Level**							0.22
Higher purchasing power	32	31.7 (0.4-82.4)	43	38.9 (20.3-61.5)	34	29.3 (0.9-62.5)	
Lower purchasing power	73	33.5 (31.2-35.8)	65	29.2 (14.3-50.6)	80	37.3 (22.7-54.7)	
**BMI***							**<0.01†**
Normal Wheigt	88	36.8 (34.3-39.4)	105	43.6 (21.5-68.6)	44	19.6 (4.9-54.2)	
Overweight/Obesity	17	22.3 (0.0-91.6)	4	4.8 (0.1-18.5)	70	72.9 (72.7-98.9)	
**Sexual Maturation**							0.37
Prepubescent	13	35.9 (22.8-51.7)	10	33.1 (11.7-64.8)	11	30.9 (17.0-49.4)	
Pubescent	74	34.8 (20.3-52.8)	75	32.9 (11.6-64.6)	71	32.3 (21.2-45.8)	
Post pubertal	18	25.6 (0.0-99.3)	23	31.2 (9.3-66.5)	32	43.4 (26.5-95.6)	
**Diet Inadequate**							0.45
Adequate	17	32.6 (9.9-67.9)	15	28.1 (22.2-34.7)	23	39.3 (17.5-66.5)	
Inadequate	88	32.9 (19.7-49.6)	93	33.4 (15.8-57.2)	91	33.6 (27.2-40.8)	
**Physically Active**							0.39
Physical activity	10	28.6 (9.7-60.0)	11	32.1 (11.7-62.8)	17	39.3 (6.1-86.5)	
Physically inactive	95	33.5 (18.9-52.1)	97	32.6 (13.3-60.3)	97	33.9 (26.3-42.6)	
**Smoking**							0.13
Not exposed	91	34.3 (19.2-53.5)	92	33.6 (19.6-51.3)	88	32.0 (29.8-34.3)	
Exposed	14	26.2 (9.4-54.8)	16	27.4 (8.3-61.4)	26	46.4 (36.0-57.1)	
**Alcohol consumption**							0.39
Not exposed	67	33.8 (24.1-45.2)	67	34.3 (27.8-41.5)	63	31.8 (26.8-37.3)	
Exposed	38	31.5 (10.2-65.1)	41	29.8 (6.9-70.9)	51	38.7 (30.9-46.9)	
**Inadequate Diet x Physically inactive ***							0.88
Adequate	1	21.6 (2.5-99.9)	2	31.2 (0.0-99.9)	3	47.2 (0.0-99.9)	
Inadequate	104	33.1 (19.2-50.7)	106	32.5 (17.3-52.7)	111	34.4 (30.9-38.0)	
**Inadequate Diet x Smoking**							0.79
Adequate	16	36.2 (35.8-89.7)	13	29.5 (17.8-44.6)	16	34.3 (5.5-82.4)	
Inadequate	89	32.3 (17.2-52.5)	95	33.0 (15.8-56.5)	98	34.6 (30.3-39.2)	
**Inadequate Diet x Alcohol consumption**							0.85
Adequate	10	31.1 (26.9-35.7)	10	33.2 (16.4-55.7)	13	35.7 (19.9-55.4)	
Inadequate	95	33.1 (16.3-55.6)	98	32.4 (14.3-58.1)	101	34.5 (31.0-38.1)	
**Physically inactive x Smoking**							0.60

Adequate	10	32.3 (16.4-53.7)	9	31.0 (8.2-69.4)	14	36.7 (5.4-85.6)	
Inadequate	95	32.9 (17.8-52.8)	99	32.7 (13.5-60.3)	10 0	34.3 (27.6-41.8)	
**Physically inactive x Alcohol consumption***							0.23
Adequate	4	19.0 (6.3-44.9)	7	38.1 (32.0-91.9)	11	42.8 (20.0-96.5)	
Inadequate	10 1	33.9 (18.1-54.4)	10 1	32.1 (12.6-60.7)	10 3	33.9 (27.1-41.6)	
**Smoking x Alcohol consumption**							0.06
Adequate	66	36.2 (26.7-46.9)	62	34.2 (25.5-44.1)	54	29.6 (27.7-31.6)	
Inadequate	39	28.9 (8.9-62.7)	46	30.5 (9.4-64.8)	60	40.7 (35.2-46.5)	
**Simultaneous adoption of unhealthy lifestyle behaviors***							0.72
1	3	34.8 (1.8-99.9)	3	24.5 (0.0-99.1)	5	40.7 (0.0-99.9)	
2	25	34.3 (15.9-59.2)	25	35.1 (29.1-41.6)	23	30.6 (15.4-51.6)	
3	50	32.3 (20.9-46.3)	47	28.9 (14.9-48.5)	61	38.7 (33.9-43.6)	
4	27	32.4 (17.9-51.2)	33	37.9 (11.9-73.3)	25	29.7 (14.4-51.4)	

Chi-square test of heterogeneity,*:Fisher's exact test, n: number or frequency, %: greater, BMI: body mass index, CI95%: Confidence interval,OR: Odds ratio, †: $p<0.05$.
